# Research culture methods detect more anaerobes compared to conventional clinical workflows in periprosthetic joint infection: a pilot study of 14 cases

**DOI:** 10.1007/s00402-026-06550-7

**Published:** 2026-10-31

**Authors:** Anabelle Visperas, Qiuhe Lu, Bethany Lehman, Suan Sin Foo, Weiqiang Chen, Vidula Vachharajani, Thaddeus Stappenbeck, Nicolas Piuzzi

**Affiliations:** 1 Department of Orthopaedic Surgery, Cleveland Clinic, Cleveland, USA; 2 Department of Inflammation and Immunity, Cleveland Clinic, Cleveland, USA; 3 Department of Infectious Disease, Cleveland Clinic, Cleveland, USA; 4 Department of Microbial Sciences in Health, Cleveland Clinic, Cleveland, USA; 5 A*STAR Infectious Diseases Labs, Singapore, Singapore; 6 Lee Kong Chian School of Medicine, Nanyang Technological University, Singapore, Singapore

**Keywords:** Periprosthetic joint infection, Culture sensitivity, Diagnostic cultures, Anaerobes, Antibiotics

## Abstract

**Introduction:**

Periprosthetic joint infection (PJI) remains one of the most challenging complications of total joint arthroplasty, often complicated by limitations in diagnostic sensitivity. Standard clinical culture methods fail to identify pathogens in 20%-50% of cases, leading to potential under- or overtreatment. This pilot study aimed to compare the microbial detection capabilities of standard clinical culture methods with enhanced research laboratory techniques, and to explore whether additional microbial findings might have the potential to influence antibiotic treatment planning.

**Materials and methods:**

Thirteen patients undergoing surgery for PJI, as defined by the 2018 International Consensus Meeting (ICM) on Infection criteria, were prospectively enrolled at a single academic medical center between August 2023 and September 2024. Intraoperative synovial fluid, periprosthetic tissue, and implant swabs were collected and processed using both standard clinical workflows and optimized research protocols. Culture results and treatment decisions were reviewed in collaboration with Infectious Disease specialists.

**Results:**

Both methods identified primary organisms. However, research techniques detected additional organisms not captured by clinical cultures (*p* = 0.013). Despite these differences, overall detection rates were comparable (57.1% vs. 59.5%; *p* = 0.963), with the research workflow achieving similar yield with fewer samples. The potential impact of additional isolate identification on antibiotic selection, if deemed clinically significant, did not appear to result in meaningful changes to antibiotic regimens, although this may have been influenced by the empiric use broad-spectrum antibiotics.

**Conclusions:**

Clinical culture methods may not detect all organisms in PJI, highlighting possible limitations in current diagnostic approaches. Although this pilot study did not directly assess the impact of enhanced microbial detection on antibiotic prescribing and treatment decisions were largely unchanged, these findings suggest that a more comprehensive microbial detection could potentially inform antibiotic selection and warrants further investigation.

## Introduction

Total joint arthroplasty is a widely performed surgery for treating end-stage osteoarthritis and other joint ailments [1]. While the majority of patients experience a complication-free improved quality of life, 0.5%-1.4% of primaries and 18%-30% of revisions develop a periprosthetic joint infection (PJI), a severe complication with profound consequences [2–7]. PJI can lead to debilitating joint dysfunction, inability to walk, amputations and a 5-year mortality rate of 11.0%-13.3% in primary cases [8, 9]. With a current case load of more than 50,000 cases per year costing over $1.6 billion in the United States, PJI is projected to increase by 200% and cost $1.9 billion by 2030 [10–13]. These alarming trends highlight the critical need for advancing diagnostic and treatment strategies to mitigate the growing impact of PJI.

Diagnosis of PJI is not straightforward in many cases and requires a battery of non-specific inflammatory tests to confirm diagnosis [14]. One major issue is that microbial detection and identification does not always yield results where it is estimated that up to 20%-50% of PJI are culture-negative even with clear clinical signs of infection [15, 16]. This may be due to multiple factors including the metabolic state of the microbe, concurrent antibiotic use, sampling error, or culturing techniques [16–18]. Biofilm-associated microbes are sessile and better protected than their free-floating planktonic counterparts, making them more difficult to culture, especially in chronic infections [17, 19]. The diagnostic workup to rule out PJI typically begins with minimally invasive sampling, including blood and synovial fluid analyses, with the latter assessed for microbial growth. Intraoperatively, synovial fluid and multiple periprosthetic tissue specimens are collected for microbiological evaluation. Of note, swabs or sonication of the explant are not standard procedures due to its processing complexity, possibly lower specificity and sensitivity compared to tissue cultures, and its use is dependent on individual institution and care pathways [20–26]. Culture procedures are not standardized across microbiology laboratories where minor methodological variations may influence microbial growth.

Treatments ranging from debridement, irrigation, antibiotics, and explantation are guided by factors such as time since index surgery, comorbidities, implant stability, and pathogen identification [27]. Microbial identification, obtained either preoperatively or intraoperatively, can dictate antimicrobial therapy [14]. In culture-negative cases, broad-spectrum antibiotics are often used empirically [12]. Antibiotic selection and management are overseen by Infectious Disease specialists, who monitor drug levels and toxicity. Notably, key aspects of antibiotic therapy, including antibiotic type, route, and duration, are not standardized across institutions and regions [28].

### Rationale

In this study, we compared the microbial detection capabilities of a state-of-the-art clinical laboratory (CL) workflow compared to research laboratory (RL) workflow to explore whether current CL workflow detect the full spectrum of microbes present in PJI. We also explored whether additional microbial findings identified by RL workflow might have had the potential to influence antibiotic treatment plans.

We hypothesize that RL culture techniques will identify a broader range of microbes than standard culture methods. The study was designed to provide preliminary evidence regarding the potential clinical implications of improved microbial detection and to inform further investigations into its impact on antibiotic decision-making and PJI management.

## Methods

### Patient population

Thirteen patients were enrolled in this single-center observational study conducted at a large academic institution from August 2023 to September 2024. Patients ≥ 18 years undergoing surgical intervention for PJI, defined by the 2018 International Consensus Meeting (ICM) on Infection criteria [14] were eligible. Exclusions included autoimmune disease or active viral infections. A single patient had repeat sample collection due to persistent infection requiring additional surgery for infection. Eight out of the fourteen cases had discontinued antibiotics at least two weeks prior to surgery, one discontinued antibiotics 1.5 weeks before surgery, and five remained on antibiotics at time of surgery. Clinical data were collected through manual chart review. 

### Sample collection (Table 1)

**Table 1:** Bacterial isolate identification workflow

	Clinical Lab (CL)	Research Lab (RL)
Sample Collection	Synovial Fluid: specimen cup for aerobic, ACT II vial for anaerobic	Synovial Fluid: purple top Vacutainer tube
	Tissue: specimen cup without media	Tissue: specimen cup with Ringers
	No. samples collected: 3–6	No. samples collected: 1
	Implant: specimen containers without media	Implant swab: specimen containers without media
Sample Collection to Received/Processed	107.2 ± 55.8 min (Range: 27.5 min to 243.3 min)	25.6 ± 31.3 min (Range: 10.0 min to 133.3 min)
Sample Preparation	Synovial Fluid: 100µL directly inoculated	Synovial Fluid: 100µL directly inoculated
	Tissue: 1–2 g tissue homogenized using a single use grinder with saline v/v, 1 drop of homogenate inoculated	Tissue: 200 mg tissue homogenized using Lysing Matrix D tube in 1 mL PBS, 100µL homogenate inoculated
	Implant: gross analysis only	Implant swab: directly
Sample Culturing	Routine: sheep blood agar, chocolate agar, and thioglycolate broth	Agar: CDC anaerobic blood agar in duplicate
	Anaerobic: CDC agar	Duplicate split into aerobic and anaerobic incubation
Culturing Conditions	Aerobic: 35–37 °C in room atmosphere supplemented with 5% CO_2_	Aerobic: 37 °C in room atmosphere
	Anaerobic: 35–37 °C with brief aerobic conditions during growth assessment	Anaerobic: 37 °C with low hydrogen mixed gas. Chamber with viewing area to maintain anaerobic conditions during growth assessment
Culture Time	Synovial fluid: 5 days (aerobic); 14 days (anaerobic)	All samples: 7 days (aerobic), 10–14 days (anaerobic)
	Tissue: 3 days (aerobic); 14 days (anaerobic)	
Bacteria Isolate Identification	MALDI-TOF (bioMerieux VITEK MS or Bruker MALDI Biotyper)	Based on morphology
		Manually picked - ~3 colonies/morphology
		Primary: Sanger sequencing full-length 16 S rRNA
		BLASTN (rRNA/ITS Databases)

Samples were collected intraoperatively under sterile conditions and stored at ambient temperature. CL specimens reached the in-house lab in 107.2 ± 55.8 min. RL specimens reached the research lab in the same institution within 25.6 ± 31.3 min (*p* = 0.0001).

#### Synovial fluid

Synovial fluid was collected before capsule incision. CL samples were divided for aerobic (specimen cup) and anaerobic [ACT II vials (Thermo Scientific, Waltham, MA)] culture. RL samples were collected in 10.8 mg K2 EDTA Vacutainer (BD, Franklin Lakes, NJ).

#### Tissue

CL samples included 3–6 knee joint specimens were collected into specimen cups. RL collected one 1cm^2^ piece of suprapatellar fat pad tissue into a specimen cup covered with Lactated Ringer’s Solution.

#### Implant

For CL, only gross implant analysis was ordered clinically. For RL, two sterile cotton tipped applicators (Medline, Northfield, IL) were used to swab the femur, tibia, and insert

### Sample preparation (Table 1)

CL at the institution is College of American Pathologists (CAP)-accredited and Clinical Laboratory Improvement Amendments (CLIA)-certified. RL, housed in the research institute, practices aseptic technique and complies with the Institutional Biosafety Committee to ensure proper handling, disinfecting, sterilizing, etc. in relation to infectious agents. All reagents are properly stored and used according to the manufacturer’s instructions.

#### Synovial fluid

For CL and RL, 100µL sample was directly inoculated.

#### Tissue

For CL, 1–2 g of tissue was homogenized using a 15mL tissue grinder (Cardinal Health, Dublin, OH) with v/v saline. One drop of homogenate was inoculated. For RL, 200 mg of tissue in 1mL PBS was homogenized using a Lysing Matrix D tube with the FastPrep-24 5G (kept at 4 °C) (MP Biomedicals, Irvine, CA). 100µL of homogenate was inoculated.

#### Implant swab

Swab was directly inoculated.

### Sample culturing, conditions, and time (Table 1)

For CL, samples were inoculated onto BD GC II Agar with Hemoglobin and IsoVitaleX Chocolate Agar plate (BD, Franklin Lakes, NJ) and BD Trypticase Soy Agar II with 5% Sheep Blood plates (BD, Franklin Lakes, NJ) or BD BBL CDC Anaerobe 5% Sheep Blood Agar plate (BD, Franklin Lakes, NJ) for aerobic and anaerobic incubation. Leftover was inoculated into thioglycolate broth enriched with vitamin K1 and hemin (BD, Franklin Lakes, NJ). Aerobic conditions included temperature between 35 and 37 °C, high humidity, and room air supplemented with 5% carbon dioxide. Anaerobic conditions included use of anaerobic canisters or envelopes in 35–37 °C incubators with brief aerobic conditions during growth assessment. Culturing time for aerobic was 3–5 days and anaerobic was 14 days.

For RL, samples were inoculated onto CDC Anaerobic Blood Agar (Anaerobic Systems, Morgan Hill, CA) in duplicate for aerobic and anaerobic incubation. Aerobic conditions included temperature set to 37 °C with standard room humidity. Anaerobic conditions include use of a vinyl anaerobic chamber (Coy Lab Products, Grass Lake, MI) with the following conditions: 37 °C with low hydrogen anaerobic mixed gas (10% carbon dioxide, 85% nitrogen, 5% hydrogen) with viewing window to maintain anaerobic conditions during growth assessment. Culturing time for aerobic was 7 days and anaerobic was 10–14 days.

### Bacteria isolate identification (Table 1)

For CL, single colony was picked and analyzed using MALDI-TOF (bioMérieux VITEK MS, Marcy-l’Étoile, France or Bruker MALDI Biotyper, Billerica, MA). If unable to confidently identify using MALDI-TOF, 16 S rRNA sequencing was used.

For RL, three colonies per morphology were manually picked and Sanger full-length 16 S rRNA sequencing was used with the BLASTN (rRNA/ITS Databases) for identification.

### Antibiotic susceptibility testing

Bacterial isolates from CL underwent antibacterial susceptibility testing using automated commercial susceptibility instruments including Vitek 2, BD Phoenix, Sensititre lyophilized BMD and/or disk/gradient strips including Etest and Liofilchem MTS. Anaerobic testing was performed at a reference laboratory. Results were used by Infectious Disease and Orthopaedic Surgery to create an antibiotic regimen. Antibiotic susceptibility testing was not completed for RL isolated bacterial isolates.

### Data analyses

Non-parametric Wilcoxon matched-pairs signed rank test with exact *P*-value, Friedman test, and McNemar’s test were calculated using GraphPad Prism 8.0 and GraphPad QuickCalcs (San Diego, CA). A *P*-value of ≤ 0.05 was considered statistically significant.

## Results

The subjects in this study with diagnosed PJI were an average age of 63 years old (range 46–80), consisted of 84.5% Caucasian and 15.5% African American patients, 61.5% were male, with an average BMI of 37.64 (range 29.33–50.84) (Table 2). These subjects had a median Charleston Comorbidity Index (CCI) of two and The American Society of Anesthesiologists physical status classification system (ASA) score of three. 69% of these subjects have had a prior PJI episode (median prior PJI interventions 1, range 0–3) with 14.3 months (range 2–38 months) since last intervention with an average of 78.8 days from symptoms to surgical intervention (range 29–150 days) (Table 2).

**Table 2:** Baseline demographics of PJI cohort

Variable	*N* (%)	Range
Age (years)	63	46–80
Sex		
Male	8 (61.5%)	
Female	5 (38.5%)	
BMI, kg/m^2^	37.64	29.33–50.84
Ethnicity		
Non-Hispanic	13 (100%)	
Race		
Caucasian	11 (84.6%)	
African American	2 (15.4%)	
Charlson Comorbidity Index (median)	2	0–6
ASA (median)	3	3–4
No. of Prior PJI Interventions (median)	1	0–3
Time to current PJI since last surgical intervention for PJI (months)	14.3	2–38
Time from symptoms to surgical intervention (days)	78.8	29–150

**Fig. 1:**
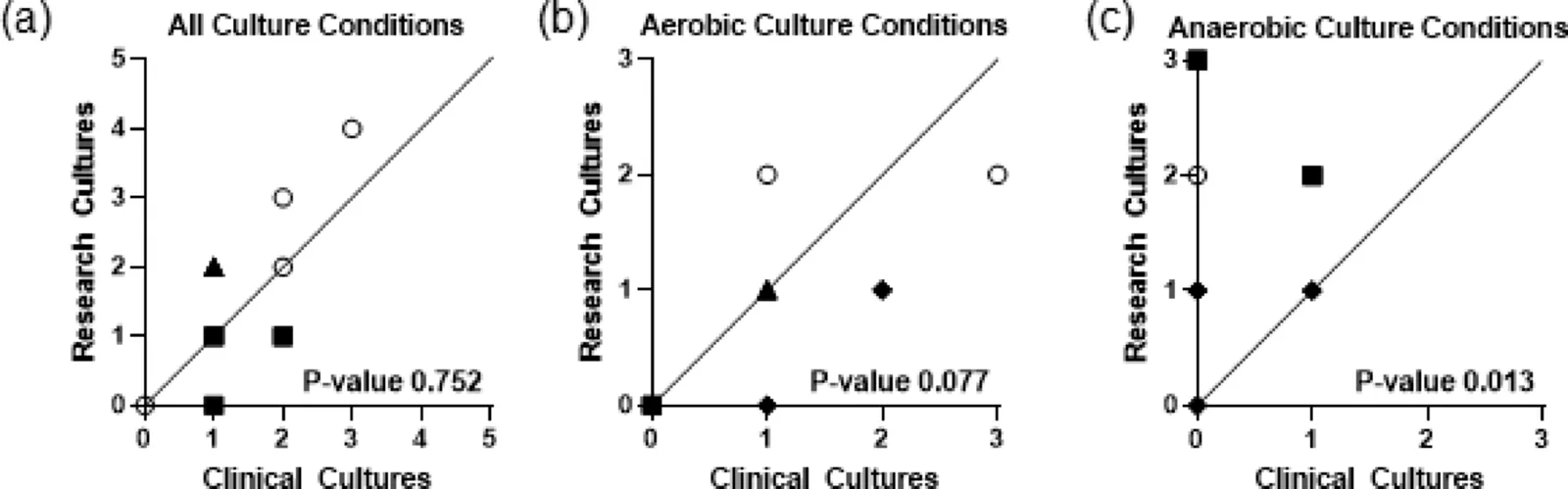
Comparison of number of culture positive samples between clinical culture methods vs. research culture methods divided by culture conditions **a** Combination of aerobic and anaerobic growth conditions; **b** Aerobic growth conditions only; **c** Anaerobic growth conditions only. Diagonal line indicates same number of microbes detected by both culture methods. Number of samples at each datapoint on graph:○ *n* = 1, ■ *n* = 2, ◆ *n* = 3, ▲ *n* = 4 (total samples in each graph *n* = 14 from 13 different patients)

Breakdown of bacterial identification in both aerobic and anaerobic culturing conditions can be found in Tables 3 and 4. Both CL and RL were able to detect 44.8% of the same microbes (13 of the total 29 detected microbes). CL methods identified seven additional unique microbes that RL methods failed to detect, specifically under aerobic culture conditions. In contrast, RL methods detected nine additional unique microbes, with 78% of these found exclusively under anaerobic culture conditions (Tables 3 and 4 and Fig. 1). Of note, six out of seven of the unique microbes detected by CL were from tissue samples (Table 3). The unique microbes detected by RL in anaerobic conditions comprised of facultative and strict anaerobes, many being gut and skin symbionts (Table 4).

**Table 3:** Aerobic culture results

ID (# prior infections)	Clinical Cultures	Research Cultures
1 (2)	*Streptococcus agalactiae* ^a, b^	*Streptococcus agalactiae* ^a, c^
2 (1)	*Staphylococcus epidermidis* ^a, b^ *Micrococcus luteus* ^b^^	*Staphylococcus epidermidis* ^a, c^
3 (1)	None^a, b^	None^a, b,c^
4 (3)	*Corynebacterium striatum* ^a^, b^	*Corynebacterium striatum* ^a^
5 (0)	None^a, b^	None^a, b,c^
6 (1)	*Staphylococcus epidermidis* ^a, b^ *Staphylococcus warneri* ^a^	*Staphylococcus epidermidis* ^b^
7 (2)	*Pseudomonas aeruginosa* ^b^	None^a, b,c^
7^&^	*Pseudomonas aeruginosa* ^b^ *Corynebacterium striatum* ^b^	*Pseudomonas aeruginosa* ^*b, c*^
8 (0)	*Streptococcus mitis*-oralis group^a, b^	None^a, b,c^
9 (0)	*Staphylococcus epidermidis* ^a^	*Staphylococcus epidermidis* ^c^
10 (0)	*Enterococcus faecalis* ^a+^	*Enterococcus faecalis* ^b, c^ *Micrococcus sp.* ^b^
11 (1)	*Cutibacterium acnes* ^a^, b^	None^a, b,c^
12 (1)	*Corynebacterium striatum* ^a, b^ *Staphylococcus epidermidis* ^a, b^ *Staphylococcus lugdunensis* ^b^	*Corynebacterium striatum* ^a, b,c^ *Bacillus (wiedmannii)* ^a^
13 (1)	*Staphylococcus lugdunensis* ^a+,b^	*Staphylococcus lugdunensis* ^a, c^

Sample type yielding positive culture: ^a^synovial fluid, ^b^tissue, ^c^implant/spacer swab

^^^Single colony reported ^&^sample taken during poly exchange

**Table 4:** Anaerobic culture results

ID (# prior infections)	Clinical Cultures	Research Cultures
1 (2)	None^a, b^	*Streptococcus agalactiae* ^a, c^ *Paraclostridium bifermentans* ^c^
2 (1)	*Staphylococcus epidermidis* ^b^	*Staphylococcus epidermidis* ^a, c^
3 (1)	None^a, b^	None^a, b,c^
4 (3)	*Corynebacterium striatum* ^a^	*Corynebacterium striatum* ^a^ *Clostridium beijerinckii* ^c^
5 (0)	*Cutibacterium acnes* ^b^	*Cutibacterium acnes* ^a, c^ *Bacillus toyonensis/Bacillus thuringiensis* ^b^
6 (1)	None^a, b^	*Staphylococcus epidermidis* ^a, b^ *Clostridium beijerinckii* ^a, b^ *Enterococcus faecalis* ^b^
7 (2)	None^a, b^	None^a, b,c^
7^&^	None^a, b^	*Corynebacterium striatum* ^b^
8 (0)	None^a, b^	None^a, b,c^
9 (0)	*Micrococcus luteus* ^b^^	*Staphylococcus epidermidis* ^a, c^
10 (0)	None^a, b^	*Enterococcus faecalis* ^c^
11 (1)	*Cutibacterium acnes* ^a, b^	*Cutibacterium acnes* ^b^
12 (1)	None^a, b^	*Corynebacterium striatum* ^a, b,c^ *Corynebacterium tuberculostericum* ^c^ *Bacteroides fragilis* ^a, b^
13 (1)	None^a, b^	*Staphylococcus lugdunensis* ^a, c^

Sample type yielding positive culture: ^a^synovial fluid, ^b^tissue, ^c^implant/spacer swab

^^^Single colony reported ^&^sample taken during poly exchange

**Fig. 2:**
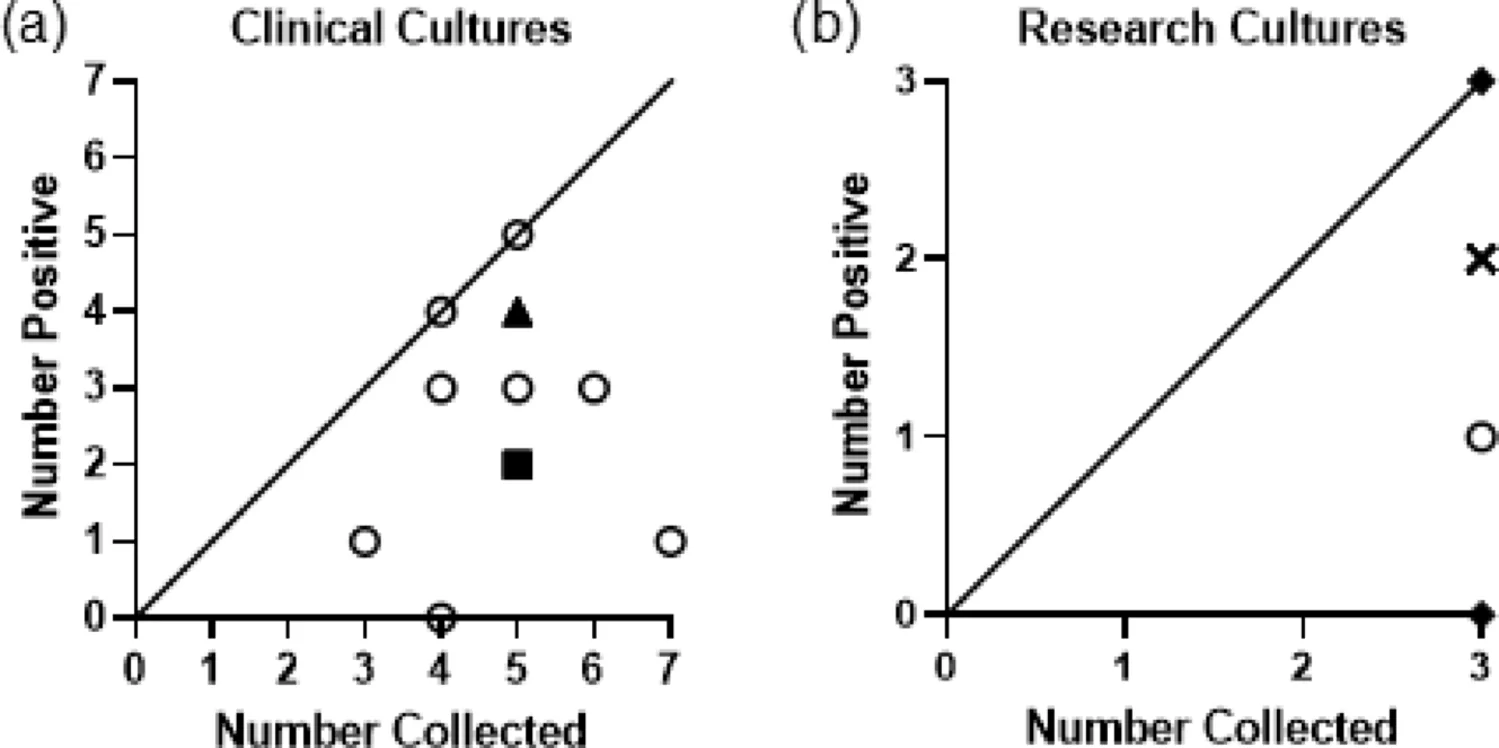
Comparison of number of cultures collected vs. number of positive cultures **a** Clinical samples collected includes synovial fluid and various periprosthetic tissues; **b** Research samples collected includes synovial fluid, suprapatellar fat pad, and swab of implant. Diagonal line indicates all samples collected yielded positive culture results. Number of samples at each datapoint on graph:○ *n* = 1, ■ *n* = 2, ◆ *n* = 3, ▲ *n* = 4, X *n* = 7(total samples in each graph *n* = 14 from 13 different patients)

Time from collection to receipt/processing was significantly longer in CL compared to RL (107.2 ± 55.8 min vs. 25.6 ± 31.3 min, *p* = 0.0001; Table 5). The number of samples collected for the differing culture methods were variable in the CL group with a median of 5 samples collected per patient (synovial fluid and various tissues, range 3–7) while RL group had 3 samples collected (synovial fluid, single suprapatellar fat pad tissue, and implant swab). Of note, a single patient only had two samples collected due to no synovial fluid available for collection. Indeed, the limited number of samples collected in the RL group yielded a similar percentage of positive cultures compared to the CL group (RL 57.1% vs. CL 59.5%, *p* = 0.963, Table 5; Fig. 2). Furthermore, when analyzing the sample types collected, for CL, a median of 4 tissue samples yielded 2.5 positive cultures (53.5% culture positivity) while RL collected a single sample with 42.9% culture positivity (*p* = 0.552, Table 5). Including swab samples into the analysis, which is absent in standard surgical procedures, culture positivity rate in RL improved to 53.6% (*p* = 0.906, Table 5).

**Table 5:** Culture methods efficiency

	Clinical	Research	P-value
Time			
Collection to received/processed (minutes)	107.2 ± 55.8 (27.50-243.3)	25.6 ± 31.3 (10.0-133.3)	*0.0001*
Sample Number			
Median no. of samples collected (range)	5^*^ (3–7)	3^+^ (2–3)	
Median no. of positive cultures (range)	3 (0–5)	2 (0–3)	
% Culture Positive	59.5	57.1	0.963

Culture Method	Median No. of Tissue Samples (Range)	Median No. of Positive Cultures (Range)	% Culture Positive	P-value
Culturing efficiency based on tissue sample number				
Clinical	4 (2–6)	2.5 (0–4)	53.5	0.552
Research	1 (1)	0 (0–1)	42.9	
Research (tissue + swab)	2 (2)	1 (0–2)	53.6	0.906

^*^Samples collected – synovial fluid, tissues ^+^samples collected – synovial fluid, tissue, swab of implant; single patient was absent of synovial fluid

**Fig. 3:**
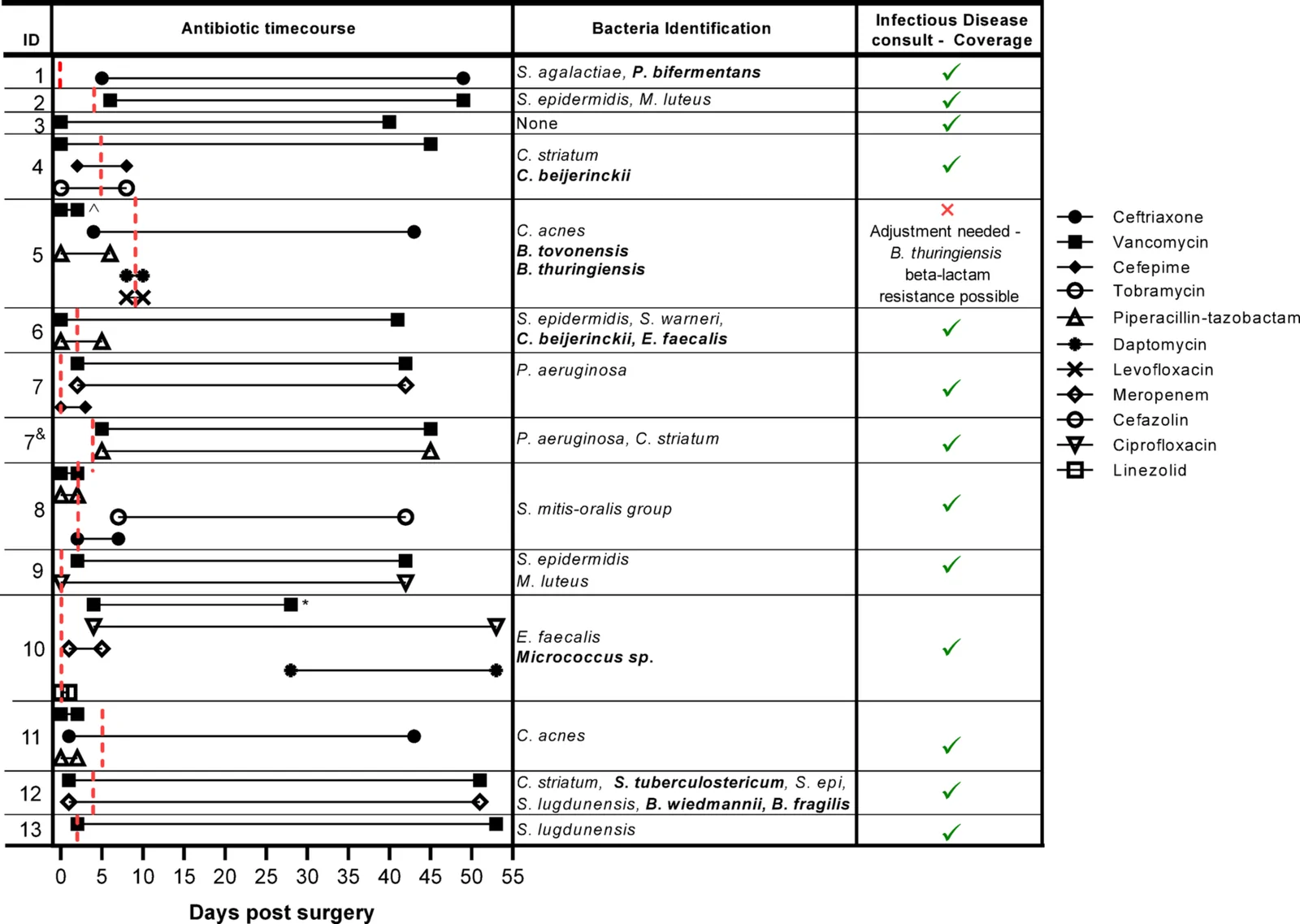
Antibiotic course for treatment of PJI. Dashed line signifies day of bacteria identification. Bold bacteria were detected by research cultures only. ^discontinued due to acute kidney injury *discontinued due to lack of therapeutic dose maintenance. *Color not required in print*

While differences in microbial detection were observed between culture methods, the potential implications for antibiotic selection were not clearly demonstrated, as theoretical treatment decisions were largely unchanged in the setting of broad-spectrum vancomycin use as the final antibiotic regimen (Fig. 3). Notably, with the added microbes detected by RL, treatment course may have been adjusted to account for resistance to beta-lactams for one patient if *B. thuringiensis* was determined to be clinically significant (Fig. 3).

## Discussion

This is the first study to evaluate the completeness of clinical laboratory cultures while also examining the potential impact of antibacterial treatment strategies in PJI. Our findings reveal that current clinical techniques effectively identify most bacterial species but are less effective in detecting strict and facultative anaerobes. Although additional microbial findings would not have substantially altered treatment decisions in this cohort, they highlight a potential limitation of current diagnostic approaches. Whether improved detection of anaerobic organisms could influence antibiotic selection, particularly where narrower-spectrum therapy may be appropriate, remains unclear and warrants further investigation.

A substantial challenge in bacterial identification remains as there is currently a 20–50% culture-negative rate in PJI, even when there is clear clinical evidence of infection [15]. While diagnosis may not always rely on culture results due to various nonspecific inflammation-related laboratory testing parameters, bacterial identification is essential for developing a comprehensive antibiotic workup. Cultures remain the gold standard for bacterial identification and is important for determining treatment protocol while balancing accuracy, completeness, and cost-effectiveness [16]. It is recommended that antibiotic treatment discontinue more than two weeks prior to surgery to increase the likelihood of obtaining reliable intraoperative cultures and may be especially beneficial in the case of culture-negative PJI [15, 29, 30]. Of note, culture negativity was not apparent in our cohort for those with continued antibiotic therapy through surgery.

To address sampling bias and improve culture positivity, multiple types and sites are sampled during surgical intervention. In our study, RL cultures identified most microbes detected by CL cultures. Notably, 86% of the microbes detected only in the CL group were detected in tissue samples. Whether these microbes would have also been detected with additional tissue sampling in RL group is unknown. Furthermore, implant swabs showed promise, as a single tissue and swab sample had the same culture positivity rate as multiple tissue samples in the CL group (CL 53.5% vs. RL 53.6%). While swabs alone do not have high specificity and sensitivity compared to tissue cultures, their use may add value to diagnosis and organism identification in combination with other testing and cultures [23].

Tissue processing methods may influence microbial recovery. In a kidney stone study, bead mill processing, which was used by RL in our study, showed a higher microbial recovery than traditional mortar and pestle based processing used in CL [31]. Transport time was also significantly different between the two groups (CL 107.2 ± 55.8 min vs. RL 25.6 ± 31.3 min) which has potential to affect culture result positivity, time to positivity, and antibiotic susceptibility. Deirmengian et al. evaluated synovial fluid culture positivity and false positive rates with delayed transport time and reported no difference, but this study was not granular enough to assess hourly differences in transport time, only in days [32]. Several other methodological differences between the RL and CL workflows could have affected microbial recovery including sample type, number of samples collected, type of agar plates with blood supplement, organism identification method, and exposure to aerobic conditions during growth assessment, although these were all within normal limits suggested by the ESGAI (European Society of Clinical Microbiology and Infectious Diseases Study Group for Anaerobic Infections) [33, 34]. Assessing variables to optimize anaerobic yield warrants further investigation.

Interestingly, many of the anaerobes detected were gut and skin symbionts. Studies have shown that increased gut permeability and gut bacteriome dysbiosis may be associated with PJI [35, 36]. Additionally, it has been shown that the knee has a distinct microbiome, even in the absence of infection [37]. This suggests a close host-microbe relationship within the knee that may play a role in the development of PJI and needs to be further investigated.

Moreover, targeted gene amplification including polymerase chain reaction (PCR) and next generation sequencing (NGS) are newer technologies that do not require proliferation of single bacterial cells to create a colony for detection and relies on DNA present in a sample [16, 38], and can prove useful in the case of culture-negative cases. These use universal primers that are conserved over all bacterial species, for example 16 S, to detect a broad array of bacterial organisms [38]. While large amounts of data can be generated and ability to detect bacteria present even if cultures are not available, its false positive rate is still high and is currently cost prohibitive to be run on every sample. Indeed, the lack of culturable bacteria also limits antibiotic susceptibility testing.

Furthermore, it is unknown whether the microbes isolated in RL methodology are clinically significant or contaminants. Large retrospective studies evaluating correlations between PJI markers (e.g. sinus tract, ESR, CRP) and specific microbes have yielded inconsistent results, limiting definitive interpretation of the clinical significance of individual microbes to these tests [39–41]. There is currently no clinical or laboratory test that is available to definitively report if a microbe detected in CL or RL workflows are significant or a contaminant, but studies are ongoing in our lab as well as others to test this [42]. Both CL and RL methods attempted to limit contaminants by collecting synovial fluid prior to capsule incision, sterile sampling, aseptic technique in processing and plate inoculation, and in RL, sequencing of multiple colonies per morphology.

Vancomycin was used in 92% of cases, offering broad microbial coverage, including those detected solely by RL cultures. Upon culture identification, final antibiotic plan was adjusted accordingly where 71% of patients continued with vancomycin (Fig. 3). Antibiotic therapy could have been altered for subject #5 if the *Bacillus* organisms isolated by RL were deemed clinically significant. As the patient was receiving ceftriaxone, a beta-lactam antibiotic, this would theoretically provide suboptimal coverage because members of the *B. cereus* group commonly harbor beta-lactamases [43]. Interestingly, in many cases, susceptibility testing identified alternative, non-broad-spectrum antibiotics that were also effective and used in combination with vancomycin, highlighting the need for ongoing discussions about antibiotic resistance. Indeed, this was the case for subject #12, if RL isolates were deemed clinically significant. Conservative antibiotic practices observed in Europe and Oceania contrast with those in the United States, where antibiotics are often prescribed for extended periods, particularly in chronic PJI cases [28].

This study has limitations. This study consisted of a small sample size with variable numbers of microbes identified and limits the ability to draw definitive conclusions regarding differences in microbial detection or potential clinical outcomes. It is unclear if the data presented is due to small sample size or true differences between workflows. This study was completed at a single-institution, single-center at a large tertiary academic referral center, relying on in-house CL and RL testing. Clinical culturing methods and bacterial identification may vary between hospitals, affecting generalizability. In addition, the CL and RL workflows differed in multiple methodological aspects including culture conditions and processing techniques. Consequently, this study cannot determine which specific methodological factors contributed to the differences in microbial recovery and warrants further investigation into which variables are clinically significant. Furthermore, this institution often manages challenging PJI cases, including patients with prior infections, higher ASA scores, and elevated BMI, which may influence the findings [44]. The validity of cultured bacteria, whether by CL or RL, cannot be independently verified, though contamination risks were minimized through preoperative decolonization strategies including chlorhexidine (CHG) wipes and nasal *S. aureus* decolonization and proper handling technique [45, 46].

## Conclusion

This study suggests that current state-of-the-art clinical culture techniques may not detect all microbes involved in PJI, particularly anaerobic bacteria. Research laboratory workflows identified additional anaerobic organisms that were not detected by clinical methods, highlighting potential gaps in current microbial detection approaches. Although the additional isolate detection would not have substantially altered treatment decisions in this cohort, likely reflecting the frequent use of broad-spectrum antibiotics, the potential clinical implications of a more comprehensive microbial detection is indicated. These findings support further investigation into whether improved detection of anaerobic organisms could inform antibiotic selection or other aspects of PJI management.

## Data Availability

No datasets were generated or analysed during the current study.
